# Evaluation of biochemical and haematological changes in dengue fever and dengue hemorrhagic fever in Sri Lankan children: a prospective follow up study

**DOI:** 10.1186/s12887-019-1451-5

**Published:** 2019-04-01

**Authors:** Grace Angeline Malarnangai Kularatnam, Eresha Jasinge, Sunethra Gunasena, Dulani Samaranayake, Manouri Prasanta Senanayake, Vithanage Pujitha Wickramasinghe

**Affiliations:** 1 Department of Chemical Pathology, Lady Ridgeway Hospital for children, Colombo, 08 Sri Lanka; 2 Department of Virology, Medical Research Institute, Colombo, 08 Sri Lanka; 3 Department of Community Medicine, Faculty of Medicine, Colombo, Sri Lanka; 4 Department of Pediatrics, Faculty of Medicine, Colombo, Sri Lanka; 5 Dehiwela, Sri Lanka

**Keywords:** Dengue fever, Dengue haemorrhagic fever, Complete blood count, Liver transaminases, Calcium, Cholesterol, Albumin

## Abstract

**Background:**

Series of biochemical and haematological changes occur during the course of dengue infection, which vary depending on the clinical disease. The patterns of change are not well documented and identifying these patterns in children with dengue infection would help to anticipate the progression to different clinical stages thus enabling effective management.

**Methods:**

A prospective follow up study was conducted during the period of July 2013 – April 2014 at Professorial Pediatric unit, Lady Ridgeway Hospital for Children, Colombo, Sri Lanka. Children (5–12 years) admitted within the first 84 h of fever, with a clinical diagnosis of dengue infection were recruited. Children who became positive for dengue IgM were included in the final analysis. Blood was collected on admission for complete blood count, Alanine aminotransferase, Aspartate aminotransferase, albumin, cholesterol and corrected calcium. These tests were repeated at 12 hourly intervals during the hospital stay.

**Results:**

Data of 130-subjects were analyzed (Dengue fever /Dengue hemorrhagic fever: 100/30). There was a significant difference in the pattern of white cell counts, platelets and haematocrit in the two clinical groups. Both transaminase rose initially in both dengue fever and dengue hemorrhagic fever and a steep rise were seen between 8th and 9th days in hemorrhagic fever. Both albumin and cholesterol decreased significantly at the time of entering into the critical phase. According to Receiver operating characteristic curve analysis, albumin level crossing 37.5g/L (sensitivity 86.7%, specificity 77.8%) and a 0.38 mmol/L reduction in cholesterol level (sensitivity 77.3%, specificity 71.9%) between day 3 and 4 were the best predictors of entering into critical phase. Calcium levels did not show any distinct pattern.

**Conclusions:**

There is a clear difference in the pattern of change of both hematological and biochemical parameters in dengue fever and dengue hemorrhagic fever. Reduction in albumin and cholesterol levels seen between the completion of day 3 and day 4 were highly valid predictors of entering into critical phase in dengue hemorrhagic fever.

**Electronic supplementary material:**

The online version of this article (10.1186/s12887-019-1451-5) contains supplementary material, which is available to authorized users.

## Background

Dengue is a mosquito-borne infection found in tropical and sub-tropical regions around the world [1]. The global incidence of dengue infection has grown dramatically over the years leading to significant morbidity and mortality in the tropical countries [1]. Plasma leakage is the hallmark pathological feature of dengue haemorrhagic fever and timely and accurate diagnosis and management of plasma leakage phase is critical for better patient outcome [2].

Series of biochemical and hematological changes occur during the course of the illness. They could be used to identify the complications early and introduce effective management strategies thus reducing morbidity and mortality. Hematological and biochemical parameters like haematocrit, albumin concentration, platelet count and aspartate aminotransferase ratio in combination is shown to be effective in identifying patients with plasma leakage in severe dengue infection [3, 4]. Hepatic involvement of varying severity is also increasingly recognized related to dengue infection [5, 6]. Derangement of liver function tests characterized by mildly raised serum total bilirubin, increased alanine transaminase (ALT) and aspartate transaminase (AST), and decreased serum albumin is commonly seen in Dengue infection and can be useful as prognostic markers [7–9]. During the plasma leakage phase of the illness, calcium, albumin and cholesterol levels also reduce in the serum [10]. Therefore these three parameters could be used as early predictors of identifying the onset of the leaking phase. The pattern of biochemical changes in the early stages of the illness and their usefulness as predictors of different phases of the illness are not well known especially in Sri Lankan setting.

## Aim and objectives

Therefore, this prospective follow-up study was designed,

- to study the pattern of change of biochemical and hematological parameters in Dengue Fever (DF) and Dengue Hemorrhagic Fever (DHF) among Sri Lankan children.
- to evaluate their usefulness as early predictors of entry into critical or plasma leaking phase.

## Methods

Children between 5 and 12 years of age, who were admitted to Professorial Pediatric unit of University of Colombo at the Lady Ridgeway Hospital for Children Colombo, within the first 84 h of onset of fever, in whom dengue infection was clinically diagnosed according to the clinical criteria (acute onset of fever and presence of two symptoms; headache/retro-orbital pain, vomiting, arthralgia/myalgia, diffuse erythematous macular rash, positive tourniquet test, leucopenia (< 5.0 × 10^9^/L), thrombocytopenia (≤ 150 × 10^9^/L) and rising haematocrit (> 5–10% above baseline)) described by the national guidelines published by Ministry of Health Sri Lanka, were recruited to the study [10]. Usually the onset of complications is after 84 h (3 .5days) and most patients with a febrile illness would be investigated and admitted to a hospital after 48 h of onset of fever. Any child with underlying chronic diseases or on long-term medication and those with Dengue IgM antibody test conducted on day 4 and day 10 of the illness, negative was excluded. Sample size was calculated to detect a standardized mean difference of 0.75 in ALT levels in the DF and DHF groups with an α error of 0.05 and a β error of 0.2, which was 30 in each group. A study conducted among children in India (7) reported mean ALT levels of 78.7 (range 16–374) and 157.3 (range 25–481) in DF and DHF patients, hence a standardized effect size of 0.75 was expected to be detected. According to the previous statistics available in the ward, the percentage of children subsequently diagnosed as DHF was about 25% out of all admissions due to suspected Dengue infection. Therefore, it was decided to recruit 120 children (in order to have 30 children with DHF). Sampling of children with a clinical diagnosis of Dengue infection was carried out consecutively until 30 patients with DHF were recruited. The total sample recruited was 130, out of which 30 were subsequently diagnosed to have DHF. Ethical approval was obtained from the ethics review committees of Lady Ridgeway Hospital and Medical Research Institute Colombo.

Informed written consent was obtained from parent or guardian. At time of enrollment, relevant clinical and demographic information of patients were collected using a structured data collection sheet. On admission 5 ml of blood was collected from each subject for complete blood count (FBC) and 5 other biochemical investigations [Alanine aminotransferase (ALT), Aspartate aminotransferase (AST), albumin, cholesterol and calcium]. Same parameters were repeated at 12 hourly intervals during the hospital stay drawing 3 ml of blood at each time. All these investigations were carried out as part of standard care of the unit. To prevent repeated venipuncture, an intravenous cannula was inserted and kept without washing exclusively to draw blood. If sample was haemolysed repeat sample was collected. If clots were noted in the cannula a fresh cannula was inserted. All children were managed according to the national guidelines of dengue management, and all children were given calcium lactate 1 mmol per kg body weight per day as per unit policy.

Time points of illness were calculated starting from the time of onset of fever and all parameters were analyzed according to the day of illness.

Beginning of the critical phase was recognized by presence of any one or more of the following three clinical, hematological or radiological features for which each patient was closely monitored for,

- progressively rising haematocrit of ≥20% from baseline
- platelet count reducing< 100 × 10^9^/L
- radiological evidence (ultrasound scan) of selective fluid leak into peritoneal cavity or pleural space

Blood was collected into EDTA tube for FBC and acid washed plain tubes for biochemical tests. Serum was separated within 2 h of collection and analyzed immediately.

All the biochemical tests were performed using, Kone-30 Lab Prime automated analyzer in day time and Mindray chemistry analyzer at night. Cross validation between the two analyzers for AST, ALT, albumin, total calcium and cholesterol were performed and the correlations (Spearman r) were 0.99, 1.0, 0.82, 0.89 and 0.95 respectively and all were statistically significant (*p* < 0.001).

ALT (normal upper limit – 40 U/L) and AST (normal upper limit – 48 U/L) were measured by modified International Federation of Clinical Chemistry recommended methods, cholesterol (normal lower limit – 3.6 mmol/L) by enzymatic method, albumin (normal lower limit – 34 g/L) by bromocresol green method and total calcium (normal lower limit for corrected calcium – 2.2 mmol/L) by arsenazo lll method.

Serum samples were tested for serological evidence of acute dengue virus infection by IgM capture enzyme linked immunosorbent commercial assay (SD Diagnostics, Korea) at Virology department, Medical Research Institute, Colombo. Blood was collected after completion of 4^th^day of illness for dengue IgM antibody assay. If the above test was negative, it was repeated on day 10 of the illness. If both tests were negative, particular patient was excluded from the analysis. The serological sensitivity of the kit was 96.4% and specificity was 98.9%.

## Statistical analysis

All statistical computations were carried out using SPSS version 21 for Windows. Complete data on biochemical and haematological parameters were available for the entire duration of hospital stay for all 130 patients. Missing data was encountered only during the latter part of the study after about day 6 when patients (especially those with DF) were discharged from the hospital. Since these were few in number and this being a descriptive cohort, these were treated as missing data and the analysis was conducted using the available data.

Changing pattern of the liver enzymes and the other biochemical parameters, calcium, albumin and cholesterol, were described according to the time of illness and phase of illness using standard descriptive statistics. As the distribution of the data showed nonparametric distribution, median values of the test results of each day of illness were calculated and plotted against the day of illness.

Univariate receiver operating characteristic (ROC) curve analysis was done initially using individual test variables (albumin, cholesterol, WBC and platelets) to determine the cut-off values, which predicted the entry into critical phase. ROC curves were drawn for day 2.5, day 3, day 3.5, day 4 values and the difference between day 3 and day 4 values. Out of them the best curves were chosen. Multivariate ROC analysis was conducted after adjusting for potential confounding factors (age, sex, past history of dengue infection) using predicted probabilities obtained through logistic regression. There was no significant difference in the areas under the curve of univariate ROC curves and the corresponding multivariate ROC curves. Therefore, the results of the univariate ROC analysis is presented.

Associations between the haematocrit and biochemical parameters (calcium, albumin, cholesterol) were analysed using Pearson’s correlation coefficient.

## Results

During the period of July 2013 – April 2014, 136 children were initially recruited to the study. Six children were excluded from the analysis as they were negative for dengue IgM antibody. Of the 130 cases included in the final analysis, 58 (44.6%) were boys and the proportion of boys was not different in the two groups (DF 46%, DHF 40%). The mean ± SD age of the children with DHF (9.4 ± 1.9) was higher than that of children with DF (8.1 ± 2.3). Median duration of illness on admission was 3 days (IQR 2.5–3.5) and 33% were admitted on 3 days of onset while 28% were admitted following 3.5 days of onset. Mean duration of hospital stay was 6 ± 1 days. As per the criteria laid down by the Sri Lankan national guidelines on management of dengue fever and dengue hemorrhagic fever in children and adolescent [10], there were 77%(*n* = 100) and 23% (*n* = 30) patients of dengue fever and dengue hemorrhagic fever respectively.

**Fig. 1:**
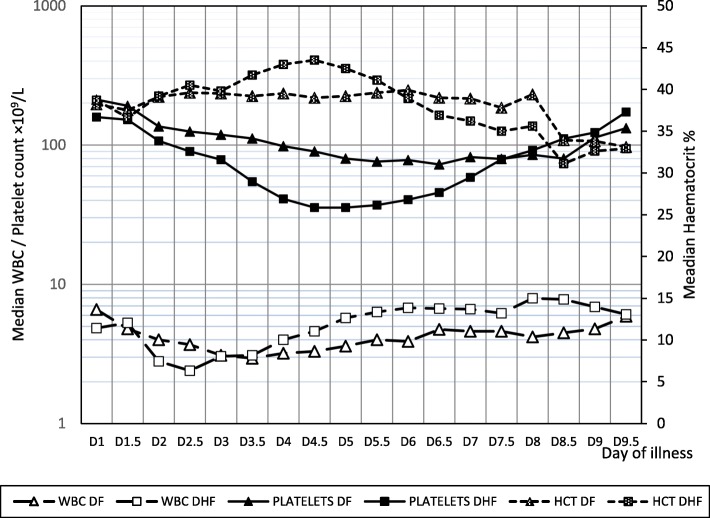
Changes in haematological parameters during the clinical course of DHF and DF in children

The pattern of hematological parameters namely the total white cell count, platelet count and haematocrit showed a marked difference between DF and DHF groups (Fig. 1). Leucopenia was more marked and the drop was steeper in DHF than in DF and the difference was statistically significant on day 2 and day 2.5. In DHF the lowest white cell count was observed around 2.5 days of illness (Median 2.4 × 10^9^/L, IQR 2.05–3.8 × 10^9^/L) and in DF it was observed around 3.5 daysof illness (Median 2.95 × 10^9^/L, IQR 2.4–3.88 × 10^9^/L). Platelets dropped later than the white cells in both DF and DHF. Platelet count dropped below 100 × 10^9^/L on day 2 of illness in DHF and day 4 of illness in DF. Lowest platelet counts were observed on day 4.5 in DHF (Median – 35 × 10^9^/L, inter quartile range (IQR) 25.75–44.28 × 10^9^/L) and on day 6.5 in DF (Median − 72.5 × 10^9^/L, IQR 55.0–97.25 × 10^9^/L). DHF had a significantly lower platelet count from day 2 to day 7. The increase in haematocrit closely reflected the decline of the platelet count in both DF and DHF. In DHF the rise in haematocrit was more distinct and rapid and was significantly higher than that of DF from day 3.5 to day 5.5. The highest haematocrit of DHF was seen on day 4, which denotes the onset of critical phase.

**Fig. 2:**
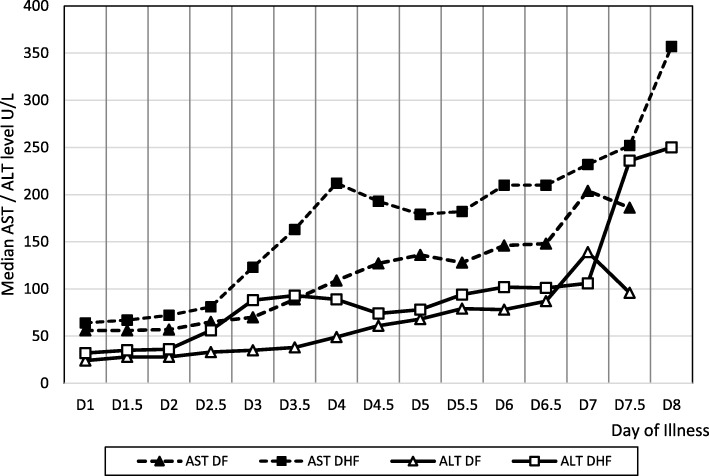
Changes in AST and ALT levels during the clinical course of DF and DHF in children

Serial changes in the transaminase levels in both DF and DHF groups are shown in Fig. 2. AST and ALT levels began to rise in the early febrile phase. Both enzyme levels increased significantly between day 3 and 4 and reached peak concentration during the later stages. Median concentration of AST at the peak was 746 U/L (IQR 215–1011 U/L). Median concentration of ALT at the peak was 118 U/L (IQR 110–314 U/L). Serum AST levels remained higher than ALT levels throughout the illness in both groups. None of the patients developed hepatic failure.

**Fig. 3:**
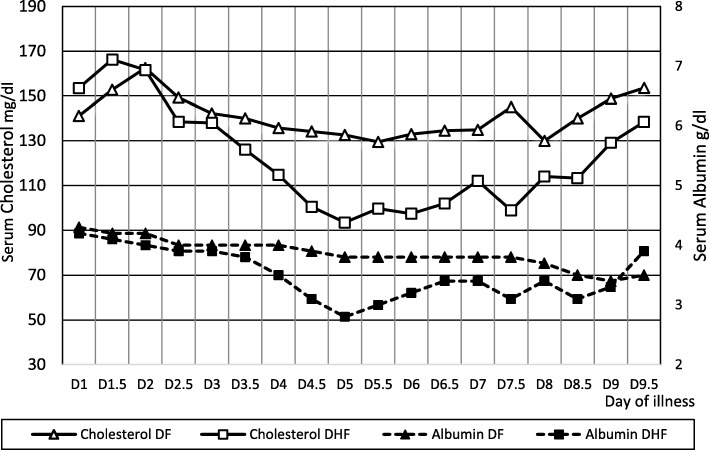
Comparing changes in Serum Albumin and Cholesterol levels in DF and DHF

In DHF serum albumin and cholesterol showed a decline with the increase in the haematocrit (Fig. 3), but these changes were not prominent in DF (Fig. 3). In DHF patients, serum albumin showed a negative correlation with the haematocrit from day 3 to day 6. This correlation was statistically significant on day 4 (r = 0.49, *p* = 0.006) and day 4.5 (r = 0.41, *p* = 0.022).

**Fig. 4:**
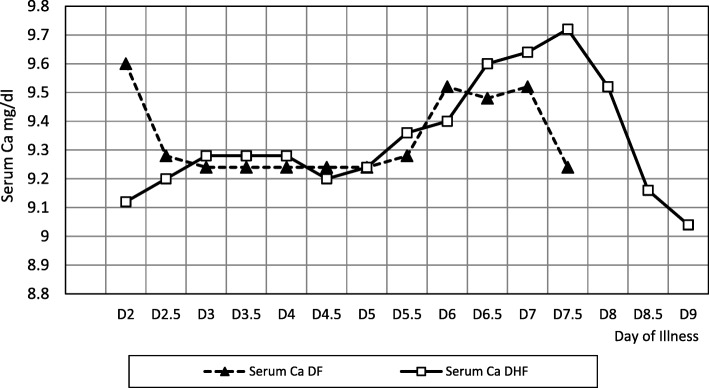
Changes in serum corrected calcium with the clinical course of the disease in DF and DHF

Serum calcium levels did not show a distinct pattern in either DF or in DHF (Fig. 4) and there was no clear association between serum calcium levels and the haematocrit in DHF patients. Median corrected serum calcium values were compared between DF and DHF groups and did not show a significant difference. However, all the patients were supplemented with calcium regularly from the time of making a clinical diagnosis of dengue infection, as it was the patient management policy of the unit.

**Fig. 5:**
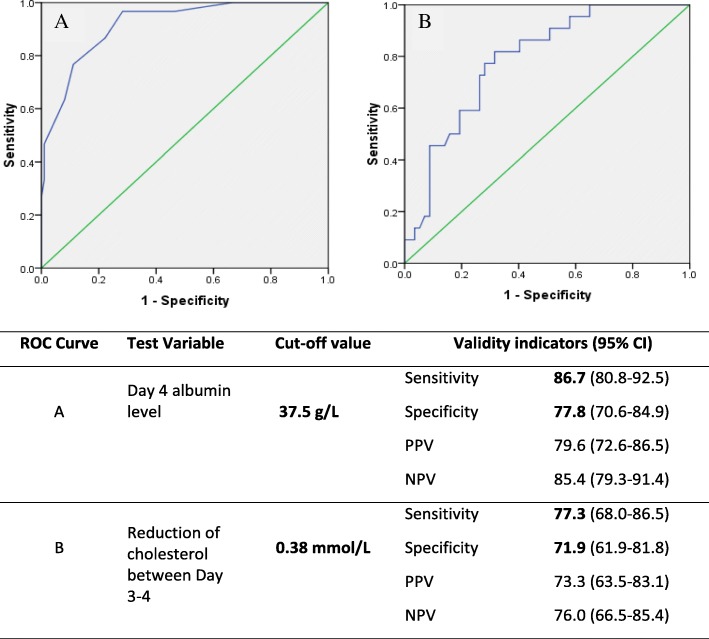
ROC curves to determine predictors of entry into critical phase. **a** - Serum albumin level on Day 4 of illness. **b** - Reduction of serum cholesterol level between Day 3 and 4 of illness

Serum albumin and cholesterol levels showed a marked decline at the time of entry into critical phase. The validity of serum albumin and cholesterol levels as a predictor of critical phase was assessed using ROC curves (Fig. 5). According to the analysis, the serum albumin levels on day 4 produced the best ROC curve with an area under the curve of 89.0% (Fig. 5A). The best cut off value of serum albumin to predict entry into critical phase was 37.5 g/L, which had a sensitivity of 86.7% and a specificity of 77.8%.

Reduction in the level of serum cholesterol was seen and values between 3rd and 4th day of illness produced the best ROC curve with an area under the curve of 78.7% (Fig. 5B). The best cut off value to predict entry into critical phase was a reduction of serum cholesterol of 0.38 mmol/L between day 3 and 4. It had a sensitivity of 77.3% and a specificity of 71.9%.

**Fig. 6:**
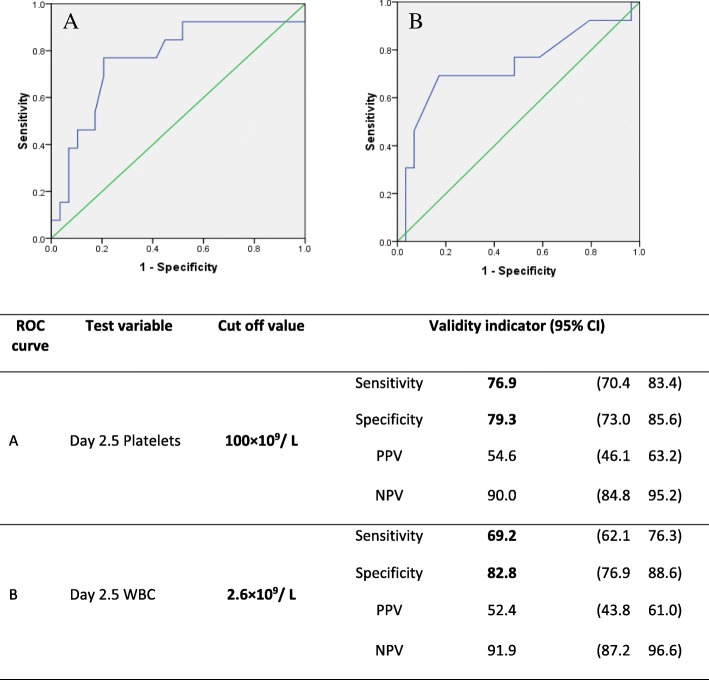
ROC curves to determine predictors of entry into critical phase. **a** – Platelet count on Day 2.5 of illness. **b** – WBC count on Day 2.5 of illness

Similarly, the validity of day 2.5 WBC count and day 2.5 platelet count as predictors of critical phase was assessed (Fig. 6). Although the predictive power was not as strong as that of serum albumin, day 2.5 platelet count of 100 × 10^9^/L had a sensitivity of 76.9% and a specificity of 79.3% in predicting entering into critical phase. Day 2.5 WBC count of 2.6 × 10^9^/L had a sensitivity of 69.2% and a specificity of 82.8% in predicting entering into critical phase.

## Discussion

Dengue infection is difficult to distinguish from the other viral infections as there are no specific clinical features that help to diagnose the disease early [11] except for polymerase chain reaction (PCR) or Non Secretary (NS) 1 antigen, which has to be done within first 48 h to have a higher yield of positive results. However still it cannot differentiate between those who progress to DF and DHF, which is determined by host of other factors that leads to significant plasma leakage in the latter part of the course of the illness. The hematological and biochemical changes that occur during the course of dengue illness could be used to predict those who are at a higher risk of developing plasma leakage and also to identify the onset of plasma leakage early. This will help the clinician to identify those who would develop DHF and have effectively managed the patients thus reducing the morbidity and mortality. Clinical spectrum of dengue virus infection has been described in detail in the past. The main focus of our study design was to identify the biochemical and hematological pattern of change and the predictors of the clinical course with the possible time of entry into critical phase.

The main hematological abnormalities were leucopenia and thrombocytopenia, caused by direct destructive action of the dengue virus. Leucopenia and thrombocytopenia were more pronounced in DHF, similar to other published results [3, 12]. A total leucocyte count of less than 2.6 × 10^9^/L and platelet count less than 100 × 10^9^/Lat day 2.5 was highly suggestive of child progressing into DHF. DHF showed a peak elevation of haematocrit during the course of illness, which correlated with the onset of leaking which occurs with the hemoconcentration due to plasma leaking.

Evidence on liver transaminases reported that elevation of AST and ALT is common in dengue infection and degree vary with severity of illness [13]. AST, rapidly rises in the early stages of the disease, especially within the first week of the illness declines gradually over next few weeks. Transaminases levels were higher in DHF than in DF and elevation of AST levels greater than the ALT levels throughout the illness, where probably former has sources other than liver, and is in agreement with the literature [9, 13]. Elevated AST levels can be used as a potential marker to differentiate dengue infection from other viral infections during the early febrile phase compared to many other common illnesses [14]. All children with DHF had elevated liver transaminases and median values were significantly higher than those with DF, a finding similar to published data [7, 9, 15]. A steep rise in transaminases during early course of illness would suggest significant liver damage which would be a deviation from the normal course of liver damage seen in dengue fever which may progress into liver failure.

From the findings of our study, albumin and cholesterol were significantly reduced at the time of entering to the critical phase. Serum albumin level less than 37.5g/L and a reduction in the serum cholesterol level by 0.38 mmol/L between 3rd and 4th days were the best predictors of entering into the critical phase. The drop in serum albumin varied with the severity of the condition and lowest level in DHF patients were seen on day 5 (30 g/dL). Previous studies showed a significant reduction in albumin and cholesterol in patients with DHF and levels are comparable to our data [3, 11, 16]. Additional significant finding in our study was the negative association between the albumin and cholesterol with haematocrit in DHF.

Hypocalcaemia occurs during the leaking phase of individuals and correction improves the outcome. Hypocalcaemia is common in DHF. Furthermore there are reports that oral supplementation reduces the disease burden [17]. In our study Calcium levels neither showed any distinct pattern nor correlation with the haematocrit. This could be due to early administration of oral calcium for all suspected cases of dengue fever. In spite of calcium therapy, there was no increase in serum calcium during the acute phase, which indicates that these patients may be leaking out calcium during acute phase and supplementation would have maintained it. This has been seen in both DF and DHF patients. Furthermore once acute phase of the illness in both DF and DHF is over there is a rise in the serum calcium level with the continuation of the supplementation. So we could postulate that there is leaking out of calcium during acute (or early) phase of the illness and once it is over the leak would have stopped with giving rise to an elevation of serum calcium with the continuation of supplementation. Case reports [17] and anecdotal evidence show that there could be drop in serum calcium levels which could be improved by early administration of oral calcium supplements. However its clinical relevance and how it would affect the natural course on the illness is not described. Control trial would be needed to find the effects of supplementation on the course of the disease.

The present study used a consecutive sample of children admitted to a tertiary care setting in Sri Lanka with clinically suspected dengue fever which was later confirmed by IgM antibody test which excluded any selection bias. Entry into critical phase being determined according to the current national guidelines on management of DF and DHF, and all haematological and biochemical tests being conducted according to standard protocols with stringent quality control, helped to minimize misclassification bias and information bias. Selected sample of cases can be considered representative of usual dengue fever patients in the tropics and the investigation-based nature of the study would make the findings of the present study highly generalizable. Focus of the current study was on comparison of biochemical and haematological changes between DF and DHF, however, if a control group with non-DF viral fever had been included, it could have added more value to the interpretation of the findings.

## Conclusion

There is a clear difference in the patterns of change of both hematological and biochemical parameters in DF and DHF. During early stages of illness, leucocyte and platelet counts could be used to predict those who would develop DHF. Drop of albumin below 37.5 g/L at day 4 and reduction of serum cholesterol level by 0.38 mmol/L between day 3 and 4 were the highly valid predictors of entering in to the critical phase.

## Additional file

Additional file 1: All data analysed during this study are included. (DOCX 18 kb)

## Abbreviations

ALT: Alanine aminotransferase
AST: Aspartate aminotransferase
DF: Dengue fever
DHF: Dengue haemorrhagic fever
EDTA: Ethylenediaminetetraaceticacid
FBC: Full blood count
IgM: Immunoglobulin M
IQR: Inter quartile range
ROC: Receiver operating characteristic
