# Supplementary material for: Evaluation of biochemical and haematological changes in dengue fever and dengue hemorrhagic fever in Sri Lankan children: a prospective follow up study
Source: BMC Pediatr. 2019 Apr 1;19:87. doi: 10.1186/s12887-019-1451-5 (PMC6442420; doi:10.1186/s12887-019-1451-5)
Supplement: Supplementary file 1 — All data analysed during this study are included. (DOCX 18 kb) [file 12887_2019_1451_MOESM1_ESM.docx]

**Additional file 1**

**Table S1: Comparison of median Serum ASTand ALTlevels between DF and DHF**

| **Day of illness** | **Median AST** | | **Sig*** | **Median ALT** | | **Sig*** |
| --- | --- | --- | --- | --- | --- | --- |
|  | **DF** | **DHF** |  | **DHF** | **DF** |  |
| 2.5 | 65.0 | 81.0 | 0.149 | 33.00 | 36.00 | 0.567 |
| 3 | **70.5** | **123.5** | **0.001** | **35.00** | **56.00** | **0.015** |
| 3.5 | **89.0** | **163.5** | **0.000** | **38.50** | **88.50** | **0.000** |
| 4 | **109.0** | **212.0** | **0.000** | **49.00** | **93.50** | **0.002** |
| 4.5 | **127.0** | **193.0** | **0.002** | **61.00** | **89.00** | **0.03** |
| 5 | **136.0** | **179.0** | **0.013** | 68.50 | 74.50 | 0.329 |
| 5.5 | **128.0** | **162.0** | **0.018** | 79.00 | 78.00 | 0.563 |
| 6 | **146.0** | **210.5** | **0.023** | 78.00 | 94.00 | 0.56 |
| 6.5 | 148.5 | 210.5 | 0.069 | 87.00 | 102.50 | 0.951 |
| 7 | 204.0 | 232.5 | 0.627 | 139.00 | 101.50 | 0.288 |

*Mann-Whitney U test

**Table S2: Comparison of median WBC, Haematocritand Platelets between DF and DHF**

| Day of illness | **WBC** | | **Sig*** | **HCT** | | **Sig.*** | **PLATELETS** | | **Sig.*** |
| --- | --- | --- | --- | --- | --- | --- | --- | --- | --- |
|  | DF | DHF |  | DF | DHF |  | DF | DHF |  |
| 1.5 | 4.8 | 5.3 | 0.72 | 37.5 | 36.6 | 0.548 | 191 | 152 | 0.26 |
| 2 | 4 | 2.8 | **0.041** | 39.1 | 39.2 | 0.6 | 136 | 107 | **0.018** |
| 2.5 | 3.7 | 2.4 | **0.016** | 39.6 | 40.5 | 0.92 | 125 | 90 | **0.006** |
| 3 | 3.1 | 3.05 | 0.5 | 39.5 | 39.8 | 0.98 | 118.5 | 78.5 | **0.000** |
| 3.5 | 2.95 | 3.1 | 0.45 | 39.2 | 41.7 | **0.018** | 111.5 | 54.5 | **0.000** |
| 4 | 3.2 | 4 | **0.024** | 39.5 | 43 | **0.000** | 98 | 41 | **0.000** |
| 4.5 | 3.3 | 4.6 | **0.005** | 39 | 43.5 | **0.000** | 90 | 35.5 | **0.000** |
| 5 | 3.6 | 5.75 | **0.000** | 39.2 | 42.5 | **0.000** | 80 | 35.5 | **0.000** |
| 5.5 | 4 | 6.35 | **0.000** | 39.6 | 41.1 | **0.03** | 76 | 37 | **0.000** |
| 6 | 3.9 | 6.8 | **0.000** | 39.9 | 38.9 | 0.3 | 78 | 40.5 | **0.000** |
| 6.5 | 4.75 | 6.7 | **0.002** | 39 | 36.9 | **0.021** | 72.5 | 45.5 | **0.000** |
| 7 | 4.6 | 6.65 | **0.006** | 38.9 | 36.2 | **0.007** | 82 | 58.5 | **0.003** |
| 7.5 | 4.6 | 6.2 | 0.106 | 37.8 | 35 | 0.26 | 79.5 | 79 | 0.604 |
| 8 | 4.2 | 7.95 | 0.071 | 39.4 | 35.6 | 0.143 | 85 | 91.5 | 1.00 |
| 8.5 | 4.5 | 7.8 | 0.5 | 33.9 | 31.1 | 1.0 | 80 | 111 | 0.5 |
| 9 | 4.8 | 6.9 | 0.5 | 33.8 | 32.6 | 0.5 | 113 | 123 | 1.0 |
| 9.5 | 5.9 | 6.1 | 1.0 | 33.1 | 32.9 | 1.0 | 132 | 172.5 | 1.0 |

*Mann-Whitney U test

**Table S3: Median values of serum Albumin, Cholesterol and corrected calcium between DF and DHF**

| **Day of illness** | **Albumin** | | **Cholesterol** | | **Serum Ca** | |
| --- | --- | --- | --- | --- | --- | --- |
|  | **DF** | **DHF** | **DF** | **DHF** | **DF** | **DHF** |
| D1 | 4.3 | 4.2 | 141.1 | 153.5 |  |  |
| D1.5 | 4.2 | 4.1 | 152.7 | 166.2 |  | 9.12 |
| D2 | 4.2 | 4 | 162.4 | 161.6 | 9.6 | 9.2 |
| D2.5 | 4 | 3.9 | 149.2 | 138.4 | 9.28 | 9.28 |
| D3 | 4 | 3.9 | 142.2 | 138 | 9.24 | 9.28 |
| D3.5 | 4 | 3.8 | 140 | 126 | 9.24 | 9.28 |
| D4 | 4 | 3.5 | 135.7 | 114.8 | 9.24 | 9.2 |
| D4.5 | 3.9 | 3.1 | 134.1 | 100.5 | 9.24 | 9.24 |
| D5 | 3.8 | 2.8 | 132.6 | 93.5 | 9.24 | 9.36 |
| D5.5 | 3.8 | 3 | 129.5 | 99.7 | 9.28 | 9.4 |
| D6 | 3.8 | 3.2 | 133 | 97.4 | 9.52 | 9.6 |
| D6.5 | 3.8 | 3.4 | 134.5 | 102 | 9.48 | 9.64 |
| D7 | 3.8 | 3.4 | 134.9 | 112.1 | 9.52 | 9.72 |
| D7.5 | 3.8 | 3.1 | 145 | 98.9 | 9.24 | 9.52 |
| D8 | 3.7 | 3.4 | 129.9 | 114 |  | 9.16 |
| D8.5 | 3.5 | 3.1 | 140 | 113.3 |  | 9.04 |
| D9 | 3.4 | 3.3 | 148.8 | 129.1 |  |  |
| D9.5 | 3.5 | 3.9 | 153.5 | 138.4 |  |  |
